# Automated detection of spontaneous calcium signaling events in prohemocytes of the *Drosophila melanogaster* lymph gland

**DOI:** 10.17912/micropub.biology.001883

**Published:** 2025-11-15

**Authors:** Xinwen Zhu, Max V.E. Smith, Guy Tanentzapf

**Affiliations:** 1 Department of Cellular and Physiological Sciences, University of British Columbia, Vancouver, British Columbia, Canada

## Abstract

Calcium signaling is an important regulator of stem cell maintenance and differentiation. Here we report the development of an image processing pipeline for *ex vivo* time-lapse microscopy data that enables the unbiased, automated detection of calcium signaling events in prohemocytes of the *Drosophila melanogaster* lymph gland. We also show that heterogeneity in gene expression driven by Tep4-Gal4, which is used to mark prohemocytes, accounts for most of the cell-to-cell variability in the signal, and that spontaneous calcium signaling events in the lymph gland can last from a few seconds to well over a minute.

**Figure 1. Automated detection of spontaneous calcium signaling events of varying duration through ex vivo time-lapse imaging of prohemocytes in the Drosophila melanogaster lymph gland:**
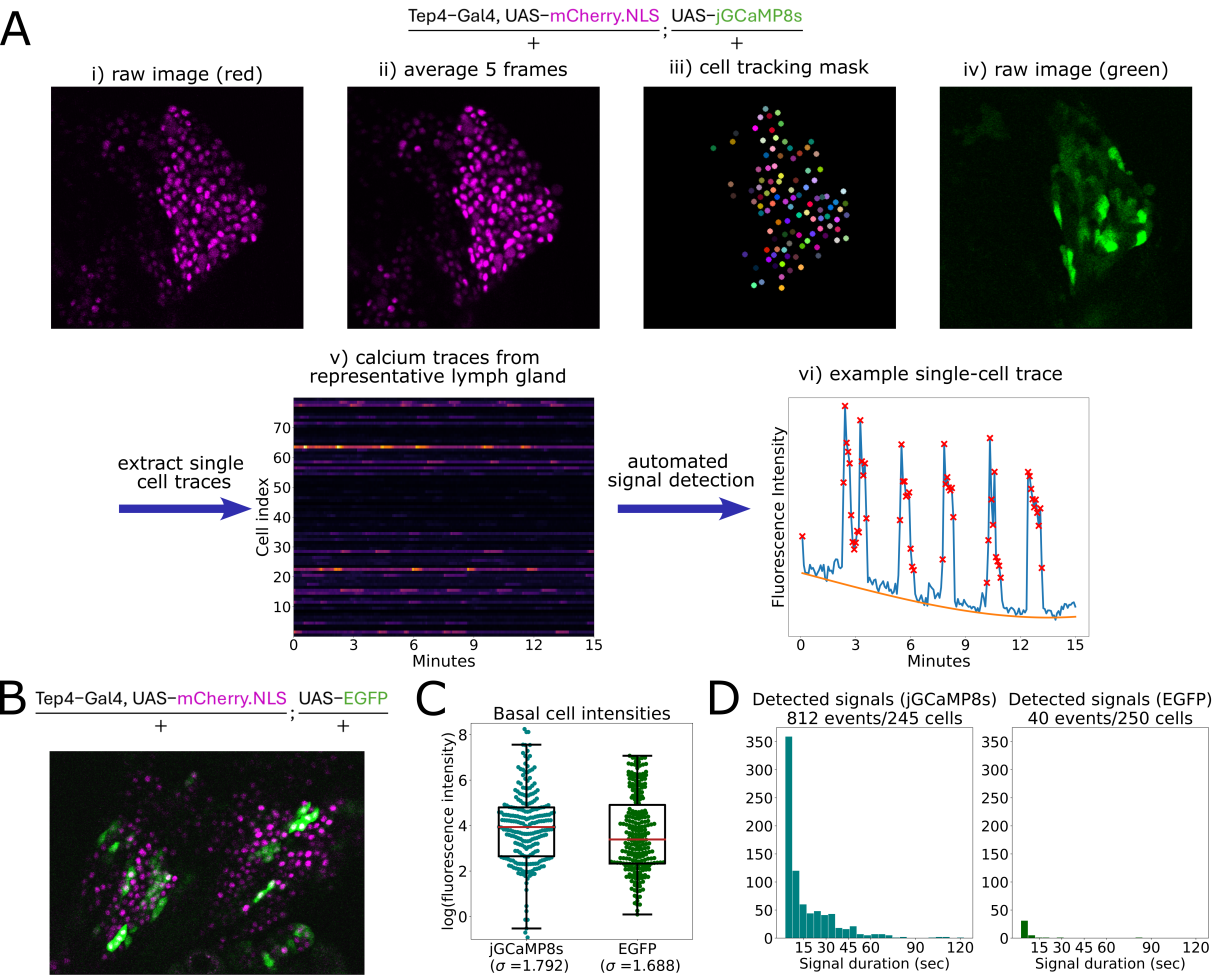
A: Overview of image processing pipeline for prohemocytes expressing nuclear-localized mCherry and the calcium biosensor jGCaMP8s using a Tep4-Gal4 driver. The raw images in the red channel (i) after photobleaching correction are averaged over every 5 frames to generate images of the cell nuclei with high signal-to-noise (ii). This time series is used to create a cell tracking mask which labels each trackable cell with a unique identifier (iii). This mask is applied to the raw images in the green channel (iv) after photobleaching correction to extract single cell traces of green fluorescence (v). Calcium signaling events are automatically detected based on relative distance to the baseline (vi, blue line = fluorescence intensity over time, orange line = estimated baseline, red x’s = time points where a signal is detected). B: Prohemocytes expressing nuclear-localized mCherry and the control fluorophore EGFP using a Tep4-Gal4 driver. C: Distribution of mean cell intensities for jGCaMP8s-expressing and EGFP-expressing prohemocytes. Box indicates the quartiles with a solid red line at the median. Standard deviations for each sample are indicated below the sample names. D: Distribution of signal durations for all detected signals in jGCaMP8s-expressing and EGFP-expressing prohemocytes. Extreme outliers (>125 seconds) were excluded from plotting. Note that since EGFP is not reactive to calcium, detected signals in EGFP samples represent false positives.

## Description

Transient changes in intracellular calcium are an important regulator of stem cell maintenance and differentiation (Tonelli et al., 2012). Our lab has previously developed methods for *ex vivo* time-lapse imaging which revealed that calcium signaling in the lymph gland is mediated by a gap-junction network that controls cell fate decisions (Ho et al., 2021). These early studies of calcium signaling in the lymph gland identified cells by manual selection of regions that display calcium dynamics. While effective, this method is labour-intensive and risks excluding cells that are quiescent or that have low signal amplitude, and biases towards cells with very clear spiking behaviour. In order to quantify global calcium dynamics in an unbiased way, we have developed a pipeline for automated detection of calcium signaling events in the lymph gland. Furthermore, we optimized a microscopy protocol to have a high image acquisition frequency (5 seconds between frames) in order to estimate the duration of calcium signaling events.

We targeted expression of the genetically-encoded calcium biosensor jGCaMP8s (Reagents et al., 2024) and a mCherry nuclear marker to the stem cell-like blood progenitors of *Drosophila* , known as prohemocytes, using the Tep4-Gal4 driver. jGCaMP8s was chosen because it is the most sensitive of the latest generation of intensiometric biosensors (Zhang et al., 2023), and its response times to changes in calcium are an order of magnitude lower than the 5 second imaging frequency that we wanted to employ. The mCherry nuclear marker was used for cell identification and tracking. Because the fluorescent nuclei are uniform in shape and non-overlapping, they are convenient for determining the locations of each Tep4+ cell in the focal plane of imaging. Furthermore, because calcium levels measured by jGCaMP8s at our imaging resolutions are fairly uniform throughout each cell at a given point in time, the average fluorescence intensity at the nucleus is a good approximation for the average fluorescence intensity of the whole cell. For cells that remained in the focal plane for the entire duration of imaging, single cell calcium activity traces were extracted and the positions at which calcium levels were higher than baseline were detected with a custom analysis script and labelled as signaling events ( Figure 1A ).

We also performed *ex vivo* time-lapse imaging on a control strain expressing the inert fluorescent protein EGFP in place of jGCaMP8s ( Figure 1B ). We had previously noticed a wide range of fluorescence intensity levels in jGCaMP8s-expressing cells and we wished to determine whether this observed heterogeneity was due to real differences in calcium levels. We calculated the basal cell fluorescence intensities (i.e. the mean fluorescence value per cell when detected signaling events are excluded) of jGCaMP8s-expressing and EGFP-expressing prohemocytes and plotted their distribution on a logarithmic scale ( Figure 1C ). Since the variability of log-transformed data is a measure of the intrinsic variability of that data (Lewontin, 1966), the standard deviations can be directly compared. While the jGCaMP8s-expressing cells have slightly higher variability in basal fluorescence (σ = 1.792) than the EGFP-expressing cells (σ = 1.688), the percent difference is only 6%. We therefore conclude that the majority of the observed heterogeneity is due to differences in Tep4-Gal4 driven gene expression rather than differences in calcium levels.

The timing of calcium signals is thought to be critically important to how they are decoded by downstream cellular processes, as sustained elevated calcium levels can impact more cellular targets (Boulware & Marchant, 2008). We therefore analyzed the durations of the detected calcium transients and found that while higher duration signaling events were less abundant than shorter ones, a third of the detected events lasted at least 20 seconds with 4% of signaling events lasting a minute or more ( Figure 1D ). The inclusion of the EGFP control strain in this analysis was useful for evaluating the performance of our automated detection, since any detected signaling events in EGFP represent false positives arising from artifacts in the imaging process. Based on the number of detected events (40 false positive events in 250 EGFP-expressing cells, and 812 total events in 245 jGCaMP8s-expressing cells), we estimate that the false discovery rate of the present experiment is under 5%. Since three-quarters of the detected signals in EGFP occur only for a single frame (duration of 5 seconds), longer detected signals are less likely to be artifacts. False positive signal detection occurs when imaging noise or tissue drift, particularly in the Z axis, causes the fluorescence intensity of a cell to change rapidly, resembling a change in intensity caused by calcium signaling. The false discovery rate can be lowered by adjusting the detection threshold, but this comes at the risk of missing real signaling events or underestimating the duration of lower amplitude signaling events. For the purposes of evaluating our pipeline in an unbiased manner, we did not remove imaging data where small amounts of tissue drift occurred as long as cells remained trackable. However, we would encourage future users to keep in mind that signal detection accuracy is related to data quality, and that manual filtering of low-quality traces may be beneficial.

Signal detection and quantification can also be improved by enhancing biosensor expression. As we have demonstrated, the Tep4-Gal4 driver used in this experiment leads to variable expression in prohemocytes, which complicates the optimization of imaging and analysis parameters. An alternative method would be to use a strong constitutive promoter such as Actin5C (Han et al., 1989) to express jGCaMP8s in all cells, and then use Tep4-Gal4 to selectively label prohemocytes with nuclear-localized mCherry. Our image processing pipeline could be applied without modifications to this alternative experimental setup and achieve more reliable signal detection. Differences in gene expression that complicate the quantification of calcium signal amplitudes can also be circumvented by transforming jGCaMP8s into a ratiometric sensor through fusion with an inert fluorescent protein that can serve as a readout for sensor levels, a strategy that has seen success in several other contexts (Farrell et al., 2024; Ichimura et al., 2021; Weigand et al., 2021).

## Methods

Strain creation and sample preparation

*Drosophila melanogaster* crosses were maintained on standard cornmeal medium (recipe available from Bloomington Drosophila Stock Center) in vials at 25°C. Experiments were performed on 3 ^rd^ instar larvae at 5 days after egg laying. Larvae were dissected and prepared for *ex vivo* live imaging as previously described (Ho et al., 2021).

Image acquisition and processing

Time-lapse imaging was performed using an Olympus FV1000 inverted confocal microscope with a numerical aperture 1.30 UPLFLN 40X oil immersion lens in a temperature-controlled chamber at 25°C. For the green channel (jGCaMP8s, EGFP), the excitation wavelength was 473 nm and emission light was collected between 490-540 nm. For the red channel (mCherry), the excitation wavelength was 561 nm and emission light was collected between 575-620 nm. For each sample, imaging was performed for 15 minutes with 5 seconds in between frames. Videos in which tissues shifted excessively or moved out of focus were excluded. Photobleaching correction was performed using the exponential fitting method of the built-in Fiji plugin (Miura, 2020). For each video, a cell identification and tracking mask was created using the Trackmate function in Fiji (Tinevez et al., 2017). First, the average of every 5 frames of the red nuclear channel was taken in order to improve signal to noise. Using the output time-averaged images, cell labels were assigned with the LoG detector and tracks were created using the overlap tracker. Finally, a cell label image was exported. A custom Python script was used to determine the green fluorescence intensities of single cells over time using the cell label image. Since each spot in the cell label image is centered on a detected nucleus and also the approximate size of an average cell nucleus, the measured green fluorescence intensities are largely comprised of nuclear signal with some surrounding cytoplasmic signal. This procedure was repeated with cells expressing only Tep4-Gal4 driven nuclear mCherry without a green fluorescent protein in order to estimate background intensity levels in the green channel. Background correction was performed before further analysis. Python packages used include Matplotlib, NumPy, and PeakUtils. Analysis code can be found on GitHub (https://github.com/Tanentzapf-Lab/Zhu_Calcium_Automated_Signal_Detection).

## Reagents

**Table:** 

**Strain**	**Genotype**	**Available from**
DGRC: 105442 (Tep4-Gal4 on chromosome II)	y[*] w[*]; P{w[+mW.hs]=GawB}NP7379 / CyO, P{w[-]=UAS-lacZ.UW14}UW14	Kyoto Drosophila Stock Center, Japan
RRID:BDSC_ 38425	w[*]; P{w[+mC]=UAS-mCherry.NLS}2; MKRS/TM6B, Tb[1]	Bloomington Drosophila Stock Center, USA
RRID:BDSC_5430	w[1118]; P{w[+mC]=UAS-EGFP}34/TM3, Sb[1]	Bloomington Drosophila Stock Center, USA
RRID:BDSC_ 605079	w[1118]; PBac{y[+mDint2] w[+mC]=20XUAS-IVS-RSET-jGCaMP8s}VK00005	Bloomington Drosophila Stock Center, USA
RRID:BDSC_64349	Canton-S (wild-type)	Bloomington Drosophila Stock Center, USA
Tep4-Gal4, UAS-mCherry.NLS; UAS-EGFP	Tep4-Gal4, UAS-mCherry.NLS/sco; UAS-EGFP/Dr	This study
Tep4-Gal4, UAS-mCherry.NLS; UAS-IVS-RSET-jGCaMP8s	Tep4-Gal4, UAS-mCherry.NLS/CyoGFP; UAS-IVS-RSET-jGCaMP8s/TM3(serGFP)	This study
Tep4-Gal4, UAS-mCherry.NLS	Tep4-Gal4, UAS-mCherry.NLS/Cyo;Dr/TM2	This study (used to determine background intensity levels)
